# Clinical Characteristics and Long-Term Outcomes of MINOCA Accompanied by Active Cancer: A Retrospective Insight Into a Cardio-Oncology Center Registry

**DOI:** 10.3389/fcvm.2022.785246

**Published:** 2022-05-20

**Authors:** Konrad Stepien, Karol Nowak, Barbara Szlosarczyk, Jadwiga Nessler, Jaroslaw Zalewski

**Affiliations:** ^1^Department of Coronary Artery Disease and Heart Failure, Jagiellonian University Medical College, Kraków, Poland; ^2^John Paul II Hospital, Kraków, Poland; ^3^Club 30, Polish Cardiac Society, Kraków, Poland

**Keywords:** MINOCA, MI-CAD, cancer, anemia, cardio-oncology

## Abstract

**Background:**

Clinical characteristics and long-term outcomes of patients with myocardial infarction with non-obstructive coronary arteries (MINOCA) and cancer are insufficiently elucidated.

**Objectives:**

We sought to characterize these patients hospitalized in a tertiary cardio-oncology center and to find the potential determinants affecting their long-term mortality.

**Methods:**

MINOCA was diagnosed in 72 of the 1,011 patients with consecutive myocardial infarction who underwent coronary angiography. Mortality rates and their determinants were analyzed within a median follow-up of 69.2 (37.8–79.9) months.

**Results:**

Active cancer was identified in 21 (29.2%) of patients with MINOCA and in 113 (12.0%) patients with myocardial infarction and obstructive coronary artery disease (MI-CAD) (*p* < 0.001). MINOCA patients with cancer were characterized by a higher incidence of anemia (47.6 vs. 21.6%, *p* = 0.03) and more frequently Takotsubo syndrome (19.1 vs. 2.0%, *p* = 0.01) than in non-cancer MINOCA. The troponin T/hemoglobin ratio was higher in both cancer MINOCA and MI-CAD groups when compared with their respective non-cancer patients (both *p* < 0.05). The age and sex-standardized mortality rates were significantly higher in cancer MINOCA (26.7%/year) when compared with non-cancer MINOCA (2.3%/year, *p* = 0.002) and in cancer MI-CAD (25.0%/year) vs. non-cancer MI-CAD (3.7%/year, *p* < 0.001). Active cancer (HR 3.12, 95% CI 2.41–4.04) was independently associated with higher long-term mortality, while higher hemoglobin levels (HR 0.93, 95% CI 0.88–0.99, per g/dl) and a MINOCA diagnosis (HR 0.69, 95% CI 0.47–0.97) improved long-term survival.

**Conclusion:**

Patients with MINOCA were comorbid with cancer more frequently than MI-CAD. In turn, an active malignancy was associated with an unfavorable long-term survival both in MI-CAD population and in patients with MINOCA.

## Introduction

Myocardial infarction with non-obstructive coronary arteries (MINOCA) is recognized if it meets the general criteria of myocardial infarction (MI) together with the absence of significant lesions in epicardial arteries in angiography (1). As shown in large MI registries, MINOCA concerns 1–13% of all patients with MI (2, 3). Recent reports indicate an unexpectedly unfavorable long-term prognosis in this group of patients. The SWEDEHEART registry included 9,092 patients with MINOCA, of whom 24% experienced a major cardiovascular event, and where 14% died within a mean follow-up period of 4.5 years (4).

The potential mechanisms responsible for MINOCA are heterogeneous (1, 5). According to the current knowledge, the underlying pathophysiological causes of MINOCA are grouped as coronary or non-coronary. Moreover, the latter are classified as myocardial disorders or as those that are typically extra-cardiac (6). Both historical (7) as well as current findings (8) indicate that hypercoagulable states, including the inherited thrombophilia, occurred in 15–25% of patients with MINOCA. This includes deficiency of protein C, protein S, or antithrombin. Additionally, the antiphospholipid syndrome was detected in 15.5% of patients. Concurrently, patients with cancer are a group that is at a particularly high prothrombotic risk, traditionally in the venous system (9). An analysis of the Surveillance, Epidemiology, and End Results involving nearly 280,000 patient pairs showed that the rate of arterial thromboembolic events was 4.7% in cancer patients compared with the 2.2% in controls (10). That predisposition for arterial thromboembolism, defined as MI, ischemic stroke, or peripheral arterial occlusion, has been confirmed recently in a large Danish population-based cohort study (1.5 vs. 0.8% in the 6-month observation, hazard ratio [HR]: 2.36, 95% confidence interval [CI]: 2.28–2.44] (11). Moreover, its occurrence among patients with cancer was associated with an increased risk of mortality (HR 3.28, 95% CI: 3.18–3.38) (11). As the arterial thromboembolic events immediately preceded cancer diagnosis and were correlated with the stage of cancer (10, 11), they can be considered paraneoplastic symptoms, which always require subsequent meticulous diagnostics toward a subclinical neoplastic process (12).

Recently, a review of the meta-regression analysis of nine studies including 26,636 patients with MINOCA has shown that 2.5% of them had a diagnosis of malignancy at presentation (13). Similar findings have been reported in the SWEDEHEART registry (14). Despite relatively low prevalence, both Nordenskjöld et al. (4) (HR: 2.40, 95% CI: 1.58–3.61, *p* < 0.001) and Pelliccia et al. (13) (coefficient: 0.001, 95% CI: −0.001 to 0.001, *p* = 0.01) have found cancer as an independent predictor of death in patients with MINOCA. Another meta-analysis including a higher number of patients with MINOCA, i.e., 36,932, did not confirm a similar relationship (15). Therefore, we sought to characterize subjects with MINOCA and cancer hospitalized in a tertiary cardio-oncology center in order to investigate the potential mechanisms affecting their long-term outcomes.

## Materials and Methods

**Figure 1:**
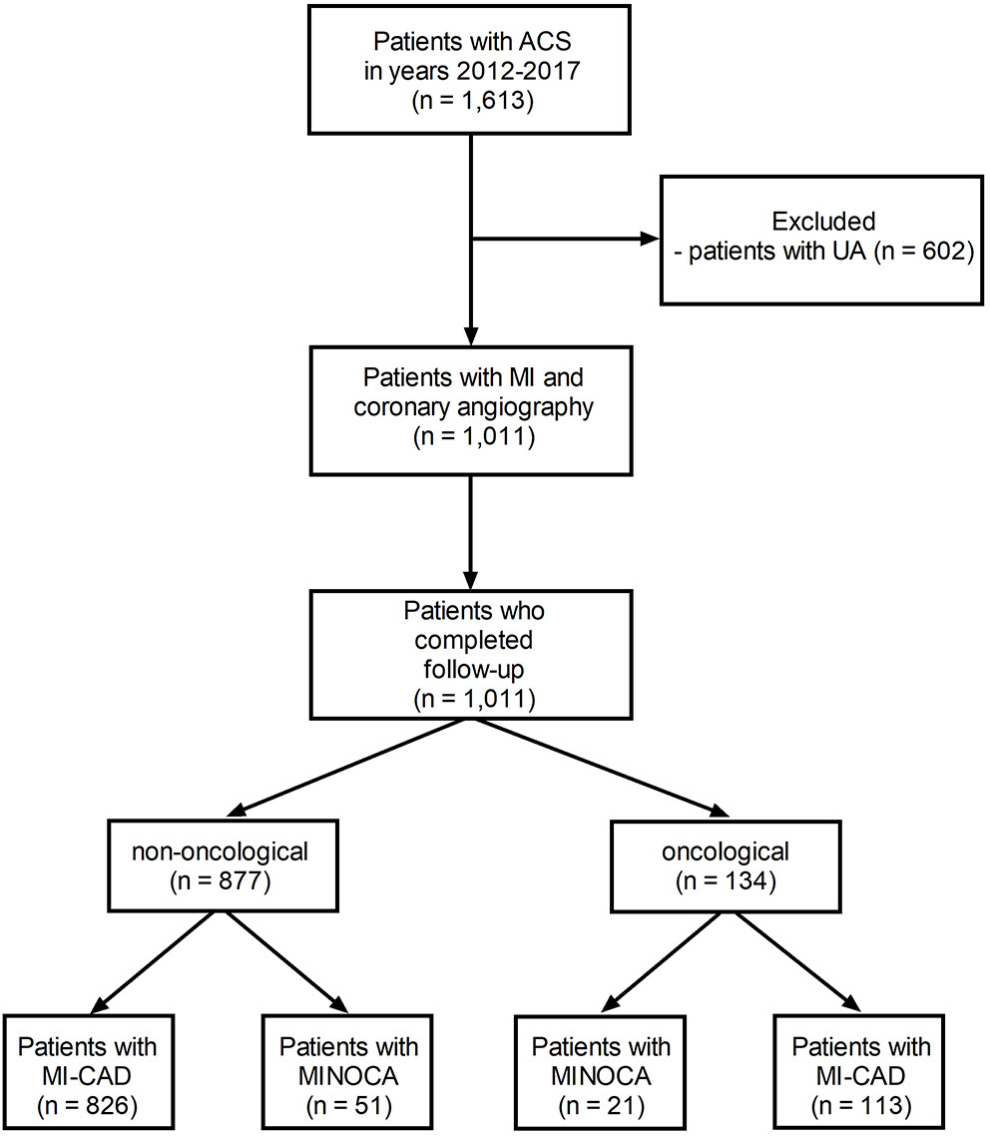
The study flow-chart. MINOCA, myocardial infarction with non-obstructive coronary artery; MI-CAD, myocardial infarction and obstructive coronary artery disease.

As has been stated retrospectively, in a tertiary cardio-oncology center including closely cooperating departments of cardiology (168 hospital beds), cardiac surgery (80 beds), pulmonology and oncology (74 beds), and thoracic surgery (48 beds), 1,011 consecutive patients underwent coronary angiography between 2012 and 2017 due to the diagnosis of MI based on clinical symptoms, electrocardiographic findings, and the evolution of myocardial necrotic biomarkers (16). MINOCA was recognized in 72 (7.1%) subjects (Figure 1) based on the universal criteria of MI (positive cardiac biomarkers rising and/or falling in serial measurements, with at least one value above the 99th percentile as the upper reference limit and at least one clinical sign of infarction). An additional inclusion criterion was a lack of obstructive lesions narrowing epicardial coronary segments by more than 50% in angiography (1, 17). Patients with ST-segment elevation of at least 1 mm in at least two contiguous leads were classified as ST-segment elevation MI (STEMI), whereas patients without ST-segment elevation on admission were diagnosed as non-ST-segment elevation MI (NSTEMI) (18). In addition, 134 (13.3%) patients were identified with active cancer, defined as cancer diagnosed within the past 6 months, receiving antimitotic treatment during the last 6 months, recurrent, metastatic, regionally advanced, or inoperable (19) (Figure 1). In the analyzed period of time, five MI patients with advanced cancer did not undergo coronary angiography and were therefore excluded from further analysis. The study protocol complied with the Declaration of Helsinki and was approved by the Jagiellonian University Medical College Ethics Committee (Consent No. 1072.6120.59.2018). All included patients gave informed consent.

### Patients Clinical and Laboratory Characteristics

Information on demographics, anthropometric parameters, cardiovascular risk factors, cardiovascular disease history, and comorbidities of all the study patients was gathered. Anemia was recognized if the hemoglobin level was <13 g/dl for men and <12 g/dl for women. The cut-off value for the thrombocytopenia was 100 × 10^3^/μl (20). Pre-end-stage renal disease and end-stage renal disease was diagnosed when creatinine clearance calculated using the Cockcroft-Gault formula was lower than 30 ml/min. Finally, creatine kinase serum activity (IU/L, upper limit of normal: 170 IU/L), isoenzyme MB of creatine kinase (IU/L, upper limit of normal: 24 IU/L), and concentration of high-sensitive cardiac troponin T (ng/ml, upper limit of normal: 0.014 ng/ml) were measured on admission and at least one time within the first 24 h.

### Angiography

All coronary angiograms were analyzed off-line, using two contralateral projections for each artery at baseline and after angioplasty if applicable, by a cardiologist unaware of the clinical data. All coronary segments were carefully evaluated for the presence of visible thrombus, distal embolization, and degree of stenosis based on visual inspection (21, 22). In cases of borderline lesions between 40 and 70%, quantitative coronary angiography (QCA Quantcor, Siemens, Germany) was applied for precise assessment. According to the guidelines (1, 5), lesions narrowing the coronary artery by <50% were defined as insignificant. All patients with insignificant stenosis were divided into two groups with either i) normal coronary arteries or minimal intracoronary irregularities with stenosis of <30% or with ii) mild to moderate lesions of at least 30 and <50%.

### Echocardiography

A two-dimensional transthoracic echocardiography was performed by a trained physician between the second and fourth day of hospitalization. It was performed at rest in a left decubitus position, using a Vivid S5 ultrasound (GE, Solingen, Germany) equipped with a multi-frequency harmonic transducer, 3Sc-RS (1.3-4 MHz). All measurements were carried out according to the recommendations of the American Society of Echocardiography and the European Association of Echocardiography (23). Standard parameters were collected to describe individual heart structures and enable their functional assessment. Screening for Takotsubo syndrome was also routinely conducted, the diagnosis of which was performed according to the InterTAK criteria (24), irrespective of the severity of coronary artery disease (25).

### Clinical Follow-Up

The length of hospitalization was collected from hospital records, whereas long-term all-cause mortality was obtained from the National Health Registry. The additional data regarding the cause of death were obtained from the Polish Office of Statistics. The causes of death were categorized as cancer, cardiovascular, other (the most common causes included respiratory system disease or accident/trauma), or unknown. Major cardiovascular causes of death included coronary artery disease, cerebrovascular disease, heart failure, or atherosclerosis.

### Statistical Analysis

Statistical analysis was performed with the SPSS Statistics software (Version 25.0.0.2, IBM, USA). Continuous variables were expressed as medians (interquartile range) and categorical variables as numbers (percentage). Continuous variables were first checked for normal distribution using the Shapiro–Wilk test. Afterward, differences in the four groups were compared with an analysis of variance, followed by a *post-hoc* Bonferroni test if the data distribution was normal. Non-normally distributed data were analyzed *via* the Kruskal–Wallis test, and differences between the groups were identified using a test for multiple comparisons of mean ranks. Categorical variables were analyzed with the chi-square test or Fisher's exact test with a *post-hoc* z-test for comparison of column proportions with the Bonferroni method. The mortality rates were expressed as crude or age and sex-standardized for the European population based on Eurostat data available online (26). The Kaplan–Meier curves for overall mortality were constructed in order to estimate the survival rates, and a log-rank test with a Bonferroni-corrected threshold was performed to assess the differences in survival between the study groups. Finally, all independent variables with the potential to confound both the exposure and the outcome were included in the Cox proportional hazard regression model to determine independent predictors of long-term all-cause mortality. A two-tailed *p*-value of <0.05 was considered statistically significant.

## Results

Based on detailed angiographic and oncological characteristics, four groups of patients were created (Figure 1). Within 1,011 MI patients, active cancer and MINOCA were identified in 21 (2.1%) patients, whereas MINOCA without cancer was diagnosed in 51 (5.0%) subjects. Of the 939 remainders with type 1 MI with obstructive coronary artery disease (MI-CAD), 113 (11.2%) patients had active cancer and 826 (81.7%) had no evidence of active cancer. In 111 patients, the malignancy process was diagnosed before index MI, whereas new cancer was found during index hospitalization in two patients with MINOCA and in 21 with MI-CAD (Table 1).

**Table 1:** Clinical and angiographic characteristics of the study patients.

	**MINOCA**		**MI-CAD**	
	**Cancer *N* = 21**	**Non-cancer *N* = 51**	**Cancer *N* = 113**	**Non-cancer *N* = 826**
Male gender	8 (38.1)	27 (52.9)	88 (77.9)	591 (71.6)
Age, years	75 (71–79)	70 (64–78)	73 (66–79)	68 (60–78)
Body mass index, kg/m^2^	24.2 (22.1–27.4)	26.7 (23.6–31.5)	26.0 (23.4–29.1)	27.7 (25.0–30.9)
Diabetes mellitus	7 (33.3)	13 (25.5)	40 (35.4)	318 (38.6)
Hypertension	16 (76.2)	47 (92.2)	96 (85.0)	717 (87.1)
Dyslipidemia	12 (57.1)	38 (74.5)	73 (64.6)	695 (84.5)
Pre-ESRD or ESRD	1 (4.8)	2 (3.9)	2 (1.8)	20 (2.4)
Active smoking	0 (0.0)	6 (11.8)	18 (15.9)	203 (24.7)
Anemia	10 (47.6)	11 (21.6)	52 (46.0)	169 (20.5)
Thrombocytopenia	3 (14.3)	2 (3.9)	3 (2.7)	9 (1.1)
Prior myocardial infarction	3 (14.3)	9 (17.7)	39 (34.5)	239 (29.0)
Prior stroke	3 (14.3)	3 (5.9)	9 (8.0)	56 (6.8)
**Killip class on admission**				
I/II	19 (90.5)	47 (92.2)	98 (86.7)	757 (91.8)
III/IV	2 (9.5)	4 (7.8)	15 (13.3)	68 (8.2)
**Clinical presentation**				
NSTEMI	15 (71.4)	45 (88.2)	74 (65.5)	530 (64.2)
STEMI	6 (28.6)	6 (11.8)	39 (34.5)	296 (35.8)
Takotsubo syndrome	4 (19.1)	1 (2.0)	0 (0.0)	8 (1.0)
Perioperative myocardial infarction	1 (4.8)		3 (2.7)	
**Type of cancer**				
Genitourinary	8 (38.1)		36 (31.9)	
Breast	5 (23.8)		6 (5.3)	
Lung	3 (14.3)		27 (23.9)	
Gastrointestinal	2 (9.5)		18 (15.9)	
Other	3 (14.3)		26 (23.0)	
**Metastatic disease**				
Lymph nodes	0 (0.0)		16 (14.1)	
Distant	4 (19.1)		24 (21.2)	
**Prior oncological treatment**				
Surgery	6 (28.6)		24 (21.2)	
Surgery with curative intent	1 (4.8)		3 (2.7)	
Radiotherapy	3 (14.3)		13 (11.5)	
Chemotherapy	4 (19.1)		28 (24.8)	
Platinum compounds	2 (9.5)		9 (8.0)	
Taxanes	2 (9.5)		2 (1.8)	
Fluoropyrimidines	0 (0.0)		10 (8.8)	
Anthracyclines	0 (0.0)		3 (2.7)	
Other	0 (0.0)		4 (3.5)	
Hormonotherapy	2 (9.5)		17 (15.0)	
Newly diagnosed cancer during hospitalization	2 (9.5)		21 (18.6)	
**Coronary angiography**				
<30% stenosis	13 (61.9)	34 (66.7)		
30–50% stenosis	8 (38.1)	17 (33.3)		
≥50% stenosis in one or two coronary arteries			87 (77.0)	687 (83.2)
≥50% stenosis in three coronary arteries			26 (23.0)	139 (16.8)
≥50% stenosis in left main			19 (16.8)	98 (11.9)
Epicardial thrombus	0 (0.0)	1 (2.0)	14 (12.4)	116 (14.0)
Distal embolization	0 (0.0)	3 (5.9)	9 (8.0)	17 (2.1)
**Treatment strategy**				
Percutaneous coronary intervention			101 (89.4)	724 (87.7)
Coronary artery bypass graft surgery			3 (2.7)	24 (2.9)
Conservative			9 (8.0)	78 (9.4)
**Pharmacotherapy**				
Aspirin	19 (90.5)	44 (86.3)	108 (95.6)	810 (98.1)
P2Y12 inhibitor	10 (47.6)	27 (52.9)	105 (92.9)	785 (95.0)
Proton pump inhibitor	8 (38.1)	35 (68.6)	84 (74.3)	618 (75.3)
ACEI/ARB	17 (81.0)	44 (86.3)	103 (91.2)	728 (88.1)
β-blocker	16 (76.2)	36 (70.6)	101 (89.4)	743 (90.5)
Statin	14 (66.7)	39 (76.5)	99 (87.6)	774 (94.3)

*Data are shown as number (percentage) or median (interquartile range), ACEI, angiotensin-converting enzyme inhibitor; ARB, angiotensin receptor blocker; ESRD, end-stage renal disease; MINOCA, myocardial infarction with non-obstructive coronary artery; MI-CAD, myocardial infarction and obstructive coronary artery disease; NSTEMI, non-ST-segment elevation myocardial infarction; STEMI, ST-segment elevation myocardial infarction*.

**Figure 2:**
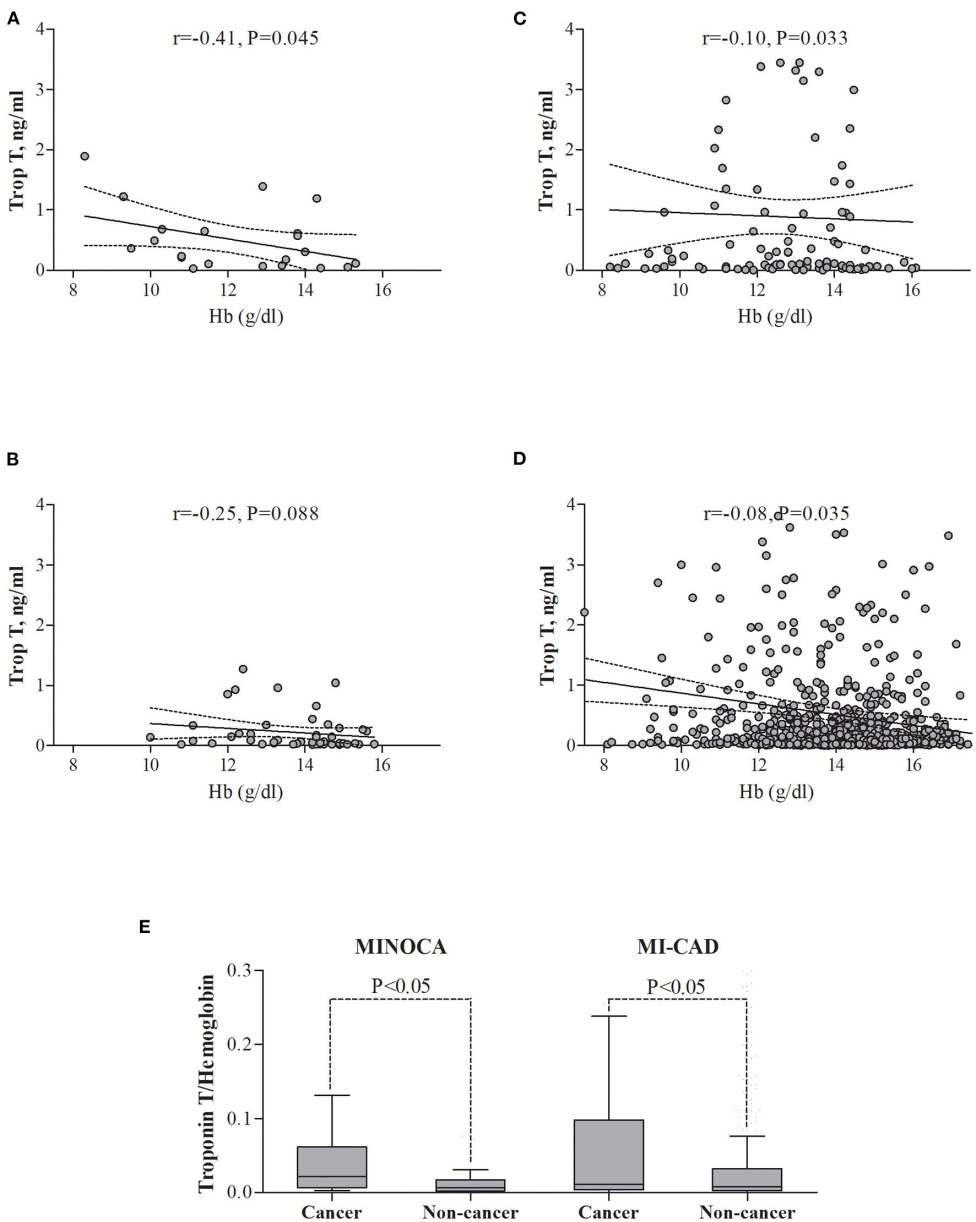
The relationships between troponin T and hemoglobin level in the study groups. **(A)** Cancer MINOCA, **(B)** Non-cancer MINOCA, **(C)** Cancer MI-CAD, **(D)** Non-cancer MI-CAD, and **(E)** In both cancer groups, the ratio of troponin T to hemoglobin was higher than in the respective non-cancer groups. MINOCA, myocardial infarction with non-obstructive coronary artery; MI-CAD, myocardial infarction and obstructive coronary artery disease.

Among the four groups, there were significant differences in the distribution of gender, anthropometric parameters, dyslipidemia, active smoking status, and initial clinical presentation (*p* < 0.01 for each) (Table 1). The angiographic analysis also revealed a different proportion of epicardial thrombus in the compared groups (*p* = 0.02). Hemoglobin levels were lower, whereas baseline high-sensitive troponin T was higher in both cancer groups compared with non-cancer MINOCA subjects (*p* < 0.05 for all pairwise comparisons) with the blurring of differences during hospitalization in maximal peak values (Table 2). After adjustment for renal function, the highest inverse correlation between hemoglobin level and baseline troponin T concentration was found in the cancer MINOCA (*r* = −0.41, *p* = 0.05) group (Figures 2A–D). The proposed ratio of troponin T to hemoglobin was higher in cancer patients with MINOCA and MI-CAD when compared with the respective non-cancer groups (Figure 2E). The time of hospitalization was insignificantly shorter in non-cancer MINOCA (4 (3–7) days) as compared with cancer MINOCA (6 (3–12) days), cancer MI-CAD (6 (3–9) days), and non-cancer MI-CAD (6 (4–8) days) and (*p* = 0.07).

**Table 2:** The selected laboratory and echocardiography characteristics.

	**MINOCA**		**MI-CAD**	
	**Cancer** ***N* = 21**	**Non-cancer** ***N* = 51**	**Cancer** ***N* = 113**	**Non-cancer** ***N* = 826**
**Laboratory tests**				
Hemoglobin, g/dl	12.9 (10.2–13.9)	14.1 (12.3–14.7)	12.8 (11.2–14.1)	14.0 (12.8–15.1)
Hematocrit, %	38.7 (31.7–41.9)	41.5 (36.6–42.8)	38.3 (34.6–41.3)	41.7 (38.4–44.6)
White blood cells, x10^3^/μl	8.9 (6.1–11.7)	8.6 (6.5–11.5)	10.0 (7.3–13.3)	9.3 (7.5–12.0)
Platelet count, x10^3^/μl	226 (166–284)	223 (163–263)	238 (182–292)	221 (184–271)
Creatinine, μmol/l	91 (76–124)	90 (73–113)	93 (77–112)	88 (76–103)
Glomerular filtration rate, ml/min	57.1 (36.7–71.2)	63.9 (53.0–88.1)	65.6 (52.7–86.0)	71.0 (57.2–86.3)
Glucose, mmol/l	7.5 (5.7–9.3)	6.3 (5.5–7.1)	7.5 (5.7–8.6)	6.9 (5.8–9.1)
Troponin, ng/ml	0.306 (0.102–0.680)	0.076 (0.027–0.265)	0.141 (0.046–1.070)	0.113 (0.033–0.429)
Troponin peak, ng/ml	0.489 (0.102–1.190)	0.145 (0.053–0.344)	0.952 (0.178–7.160)	0.897 (0.249–4.300)
Creatine kinase, IU/l	134 (51–163)	132 (90–266)	151 (82–376)	186 (109–381)
Creatine kinase peak, IU/l	137 (77–246)	150 (99–319)	313 (140–852)	553 (192–1,652)
Creatine kinase MB isoenzyme, IU/l	24 (13–35)	20 (14–29)	23 (15–61)	22 (15–45)
Creatine kinase MB isoenzyme peak, IU/l	27 (19–42)	21 (16–32)	44 (23–145)	61 (26–155)
**Echocardiography characteristics**				
Right ventricular systolic pressure, mmHg	45 (33–63)	32 (26–40)	36 (29–44)	28 (26–37)
TAPSE, mm	24 (20–28)	22 (20–25)	22 (16–24)	21.8 (19–25)
Left atrium, mm	36 (33–43)	42 (36–45)	41 (38–46)	42 (38–46)
E/A ratio	0.6 (0.5–0.8)	0.8 (0.6–1)	0.8 (0.7–1)	0.7 (0.6–1.1)
End-diastolic LV diameter, mm	45 (41–52)	50 (45–53)	51 (46–56)	51 (48–56)
End-systolic LV diameter, mm	25 (23–33)	32 (27–37)	34 (29–42)	32 (28–37)
LV ejection fraction, %	50 (40–59)	55 (45–60)	45 (36–55)	50 (40–55)
Aortic valve peak gradient, mmHg	8.5 (7–13.5)	7 (6–10)	7 (5–9)	7 (5–8)
Ascending aorta diameter, mm	34 (29–36)	36 (33–38)	35 (33–38)	36 (33–38)

*Data are shown as median (interquartile range), HDL, high-density lipoprotein; LDL, low-density lipoprotein; LV, left ventricular; MINOCA, myocardial infarction with non-obstructive coronary artery; MI-CAD, myocardial infarction and obstructive coronary artery disease; TAPSE, tricuspid annular plane systolic excursion*.

### Active Cancer Diagnosis Among MINOCA Patients

MINOCA was recognized significantly more often in cancer patients (21 of 134) compared with the non-cancer (51 of 877) cohort (15.7 vs. 5.8%, *p* < 0.001). A higher percentage of women was found in both cancer and non-cancer MINOCA groups than in the respective MI-CAD populations (*p* < 0.05 for both pairwise comparisons). A higher incidence of anemia was observed in cancer vs. non-cancer MINOCA group (47.6 vs. 21.6%, *p* < 0.05), without a significant difference in thrombocytopenia (14.3 and 3.9%). In both groups, the vast majority of MIs were classified as NSTEMI (71.4 and 88.2%, respectively). Similar treatment regimens were found in both MINOCA subgroups (Table 1). Aspirin was used in 90.5 and 86.3% of patients, respectively, whereas P2Y12 inhibitor was used in approximately half of the patients in both groups. Only proton pump inhibitors were used less frequently in cancer than in non-cancer MINOCA patients (38.1 vs. 68.6%, *p* < 0.05).

The echocardiographic screening showed more frequent Takotsubo syndrome in the oncological patients (19.1 vs. 2.0%, *p* = 0.010), with almost the same distribution of insignificant lesions in angiography in both groups (Table 1). Both epicardial thrombi and distal embolization were not found in cancer MINOCA and were reported only in the single non-cancer patients with MINOCA. Higher right ventricular systolic pressures (*p* = 0.03) and lower left atrium diameters (*p* = 0.05) (Table 2) were found in cancer vs. non-cancer patients with MINOCA with no differences in left ventricular ejection fraction (LVEF).

### Active Cancer in Patients With MI With vs. Without Obstructive Coronary Artery Disease

Active cancer was found more often in patients with MINOCA (21 of 72) compared to patients (29.2 vs. 12.0%, *p* < 0.001) with MI-CAD (113 of 939) (Table 1). Men were almost two times as represented in the cancer MI-CAD group compared with the MINOCA subgroup (77.9 vs. 38.1%, *p* < 0.05). Almost half of the patients had anemia in both groups, and both cancer groups presented with thrombocytopenia less frequently than anemia (Table 1) in a similar proportion when compared with respective non-cancer populations. In-hospital use of P2Y12 inhibitors (47.6 vs. 92.9%, *p* < 0.001), proton pump inhibitors (38.1 vs. 74.3%, *p* = 0.001), and statins (66.7 vs. 87.6%, *p* < 0.05) was less frequent in cancer MINOCA than in cancer MI-CAD.

In half of the newly diagnosed neoplasms, the first symptom was bleeding associated with antiplatelet and/or antithrombotic treatment administered during index MI, including hematuria (26%), hemoptysis (13%), and bleeds from the gastrointestinal tract (13%). Genitourinary neoplasms were predominant in both patients with MINOCA and MI-CAD (38.1 and 31.9%, respectively), whereas breast cancer was more frequent in the MINOCA group (23.8 vs. 5.3%, *p* = 0.02). There were significant differences neither in the locoregional and distant advancement of the neoplastic process nor in the anticancer treatment applied before the index MI (Table 1). The most commonly used chemotherapeutic agents in the MINOCA group were platinum compounds and taxanes. In turn, platinum compounds and fluoropyrimidines dominated in MI-CAD (Table 1).

Epicardial thrombus (12.4%) as well as distal embolization (8.0%) were observed numerically in a high percentage of cancer patients with MI-CAD but were not found in the cancer MINOCA group (Table 1). In the majority of cancer patients with MI-CAD, the significant atherosclerotic lesions were limited to one or two coronary arteries (77%). Most of these patients were treated with percutaneous coronary intervention (89.4%). In contrast, Takotsubo syndrome among patients with cancer was diagnosed only in the MINOCA group (19.1 vs. 0.0%, *p* < 0.05) (Table 1). There were no significant differences in right ventricular systolic pressure (*p* = 0.18) and LVEF (*p* = 0.28), but significantly larger end-diastolic (*p* = 0.02) and end-systolic (*p* = 0.03) left ventricular (LV) diameters were identified in the cancer MI-CAD group. Chemotherapy and radiotherapy administered before index MI did not affect LVEF (*p* = 0.59), end-diastolic (*p* = 0.90), or end-systolic (*p* = 0.86) LV diameters (Table 2).

### Long-Term Mortality, Its Causes, and Predictors

**Figure 3:**
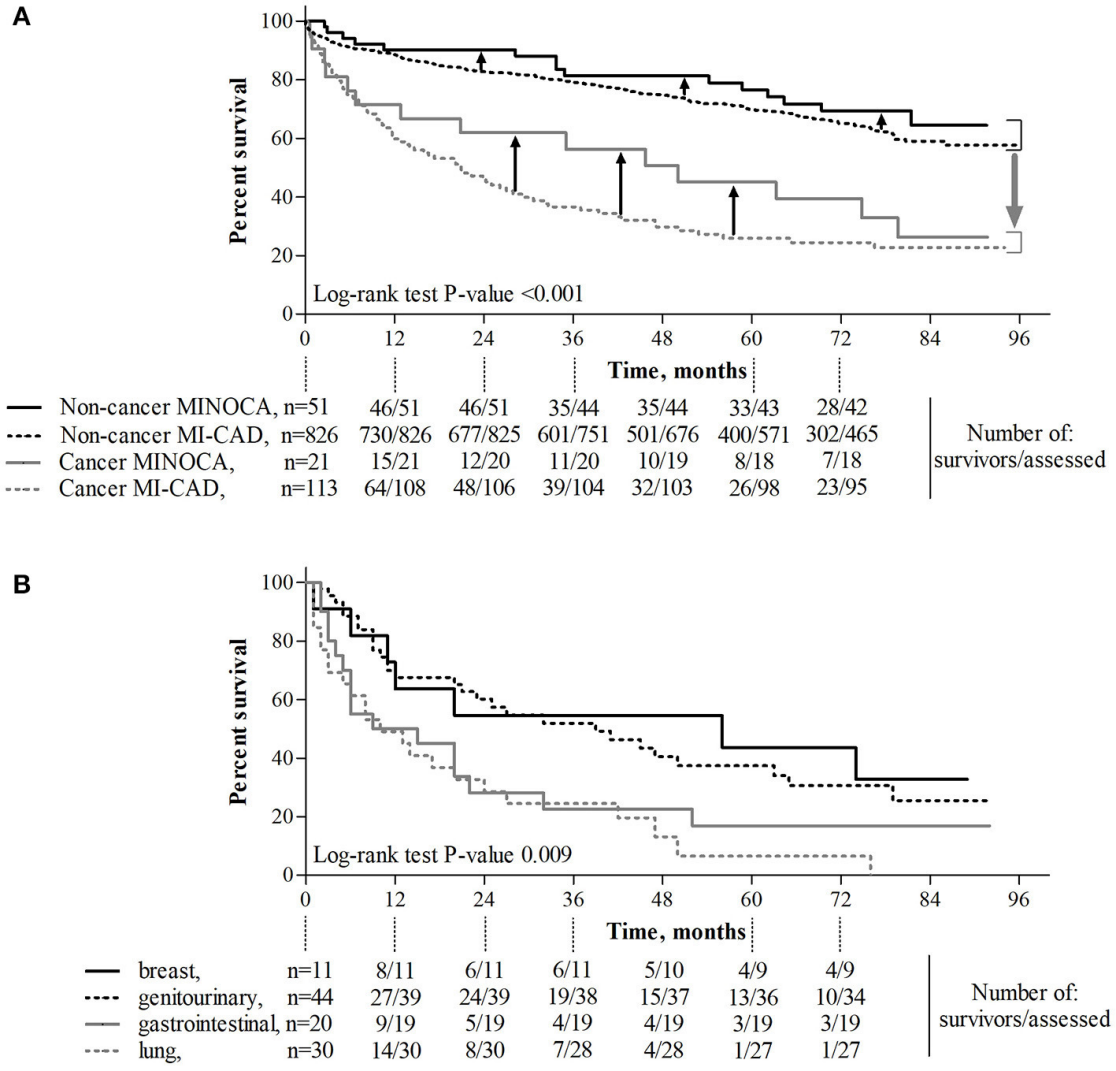
The effect of cancer and its type and MINOCA on long-term survival. **(A)** Diagnosis of cancer was associated with significantly reduced long-term survival (*p* < 0.001, gray, wide arrow), whereas MINOCA diagnosis improved (*p* = 0.048, black, narrow arrows) long-term survival. **(B)** Long-term survival in patients with lung cancer was lower than that in those with genitourinary cancer (*p* = 0.001) or breast cancer (*p* = 0.018). MINOCA, myocardial infarction with non-obstructive coronary artery; MI-CAD, myocardial infarction and obstructive coronary artery disease.

The median follow-up time in patients with non-cancer MINOCA, non-cancer MI-CAD, cancer MINOCA, and cancer MI-CAD was 73.4 [33.7–81.7], 41.9 [28.1–73.5], 35.0 [6.2–77.2], and 17.3 [4.9–43.9] months, respectively (*p* < 0.001). Both crude or age- and sex-standardized mortality rates as well as causes of death differed among the four groups (Table 3). As expected, the higher prevalence of cancer deaths was more pronounced in both oncological groups. In turn, cardiovascular causes of death were predominant in both non-cancer MINOCA and MI-CAD groups. Long-term survival was significantly higher in non-cancer MINOCA when compared with cancer MINOCA (HR 4.07, 95% CI 1.72–9.64, *p* = 0.002) and in non-cancer MI-CAD when compared with cancer MI-CAD (HR 7.62, 95% CI 5.13–11.31, *p* < 0.001). Concurrently, there were no significant differences in long-term survival between both cancer groups of MINOCA and MI-CAD (HR 0.76, 95% CI 0.45–1.28, *p* = 0.31), as well as both non-cancer groups of MINOCA and MI-CAD (HR 0.80, 95% CI 0.50–1.28, *p* = 0.35) (Figure 3A). The median survival time irrespective of the type of MI was 56, 39, 12, and 10 months for breast, genitourinary, gastrointestinal, and lung cancer, respectively (Figure 3B). A significantly better survival rate was found in patients with genitourinary cancer vs. lung cancer (HR 0.34, 95% CI 0.18–0.65, *p* = 0.001) and in breast cancer vs. lung cancer (HR 0.39, 95% CI 0.18–0.85, *p* = 0.02).

**Table 3:** The long-term mortality and its causes.

	**MINOCA**		**MI-CAD**		***P*-value**
	**Cancer** ***N* = 21**	**Non-cancer** ***N* = 51**	**Cancer** ***N* = 113**	**Non-Cancer** ***N* = 826**	
Patients who died during follow-up	14 (66.7)^#, ∧^	15 (29.4)	82 (72.6) #,∧	256 (31.0)	<0.001*
Crude mortality rate, %/year	19.2^#, ∧^	5.9	31.7^#, ∧^	7.9	<0.001**
Age- and sex-standardized mortality rate, %/year	26.7^#, ∧^	2.3	25.0^#, ∧^	3.7	<0.001**
**Causes of death, expressed as number (% of patients who died)**					
Cancer	6 (42.8)^#, ∧^	3 (20.0)	46 (56.0)^#, ∧^	45 (17.6)	<0.001*
Unknown	0	1 (6.7)	3 (3.7)	8 (3.1)	
Other	2 (14.3)	3 (20.0)	9 (11.0)	54 (21.1)	
Cardiovascular:	6 (42.8)	8 (53.3)	24 (29.3)^#, ∧^	149 (58.2)	
Coronary artery disease	1 (7.1)	2 (13.3)	8 (9.8)	63 (24.6)	NA*
Cerebrovascular disease	1 (7.1)	2 (13.3)	4 (4.9)	22 (8.6)	
Heart failure	2 (14.3)	3 (20.0)	6 (7.3)	28 (10.9)	
Atherosclerosis	2 (14.3)	1 (6.7)	6 (7.3)	36 (14.1)	

Data are shown as number (percentage) unless otherwise indicated, MINOCA, myocardial infarction with non-obstructive coronary artery; MI-CAD, myocardial infarction and obstructive coronary artery disease; NA, not applicable; p-value for differences in four groups based on a chi-square test with a post-hoc z-test for comparison of column proportions with the Bonferroni method (*) or a log-rank test for multiple comparisons of survival curves with the Bonferroni-corrected threshold (**), ^#^p < 0.05 non-cancer MINOCA, ^∧^p < 0.05 non-cancer MI-CAD.

In the MINOCA group, there were no significant differences in the long-term survival between patients with vs. without Takotsubo syndrome (Supplementary Figure 1). There was also a significantly higher long-term mortality rate in cancer vs. non-cancer patients matched for age, gender, body mass index, diabetes, hypertension, and hyperlipidemia (Supplementary Table 1 and Figure 2). A Cox proportional hazard regression limited to patients matched for demographic parameters and cardiovascular risk factors showed that unfavorable prognosis was associated with active cancer, a lower hemoglobin level, and age of older patients. Simultaneously, hypertension, hyperlipidemia, and better LVEF independently improved long-term survival (Supplementary Table 2).

In the whole group, age, female gender, cancer, anemia, and lower hemoglobin level were identified as associated with a higher mortality rate in a univariate model (Table 4). Using a Cox proportional hazard regression, an active cancer was independently associated with a higher long-term mortality rate, while higher hemoglobin levels and MINOCA diagnosis improved long-term survival (Table 4). A Cox proportional hazard regression limited to only patients with MINOCA showed that age, cancer, and LVEF were independently associated with a long-term mortality rate (Table 4).

**Table 4:** The independent predictors of death in the whole group and in patients with MINOCA.

	**Univariable model**			**Multivariable model**		
	***P*-value**	**HR**	**95% CI for HR**	***P*-value**	**HR**	**95% CI for HR**
**The whole group**						
Age, per year	0.009	1.01	1.00–1.02	0.24	1.01	0.99–1.02
Male gender, yes/no	<0.001	0.65	0.53–0.80	0.53	0.93	0.74–1.17
Active cancer, yes/no	<0.001	3.33	2.64–4.21	<0.001	3.12	2.41–4.04
MINOCA, yes/no	0.18	0.90	0.65–1.15	0.048	0.69	0.47–0.97
Anemia, yes/no	<0.001	1.76	1.40–2.20	-		
Hemoglobin, per 1 g/dl	<0.001	0.88	0.84–0.93	0.018	0.93	0.88–0.99
LVEF, per 5%	0.74	1.00	0.99–1.01	-		
Killip 3/4 vs. 0/1 on admission	0.61	1.10	0.77–1.57	0.94	1.01	0.71–1.45
**MINOCA patients**						
Age, per 1 year	0.019	1.05	1.01–1.10	0.044	1.04	1.00–1.08
Female gender, yes/no	0.73	1.14	0.55–2.37	-		
Active cancer, yes/no	0.003	3.09	1.49–6.41	0.040	2.24	1.04–4.80
LVEF, per 5%	0.007	0.96	0.94–0.99	0.012	0.95	0.93–0.97

*CI, confidence interval; HR, hazard ratio; LVEF, left ventricular ejection fraction; MINOCA, myocardial infarction with non-obstructive coronary artery*.

## Discussion

To our knowledge, this study is the first and most comprehensive analysis derived from a tertiary cardio-oncology center concerning the complex relationship between cancer and MINOCA, as well as its influence on long-term clinical outcomes. As shown, neoplasm has been identified more frequently in patients with MINOCA than in those with atherosclerosis and/or thrombus-based type 1 MI (defined as MI-CAD). However, a multivariable analysis showed that an active malignancy was associated with unfavorable long-term outcomes. We have also provided clinical features that characterized cancer patients with MINOCA, which might be useful in their differential diagnosis. It is important to note that the diagnosis of cancer in both MINOCA and MI-CAD groups was associated with an extremely high all-cause mortality in a 5-year observation. Moreover, a multivariable approach limited to only the MINOCA group showed that active cancer irrespective of age and lower left ventricular systolic function affected a higher mortality rate.

Patients with MI-CAD and cancer distinguished in our study were characterized by a highly unfavorable prognosis driven mostly by neoplastic disease. Although treatment of such patients should be strictly individualized, there are still limited data sufficiently addressing the optimal management of MI in patients with cancer (27). Further studies are warranted to establish an optimal antithrombotic regimen, especially in the acute phase, due to the proven high risk of stent thrombosis (9, 28). The results derived from the large Nationwide Inpatient Sample indicate that cancer in patients receiving percutaneous coronary intervention is common, but its prognostic impact depends on detailed oncological characteristics (29). Our results also indicate that, in both cancer and non-cancer MI-CAD patients, the rate of revascularization with the percutaneous coronary intervention was almost 90% emphasizing current trends in interventional cardiology. While cancer patients with type 1 MI were historically less likely to receive primary percutaneous coronary intervention with first-generation drug-eluting stents mainly due to the need for a shorter course of dual antiplatelet therapy following bare-metal stents, the new drug-eluting stents requiring shorter antiplatelet therapy time have become more effective and as safe as bare-metal stents. According to the current registries, dual antiplatelet therapy was prescribed in only half of the patients with MINOCA, mainly in those with sinus rhythm, prior percutaneous coronary intervention, and active smokers (30).

In contrast, the prognosis in patients with MINOCA remains controversial, with the latest studies suggesting comparable (4, 31) or lower (15) long-term mortality rates in patients with MINOCA vs. MI-CAD. The abovementioned studies indicate that a history of cancer coexisting with 2–2.5% of patients with MINOCA (4, 13) is (13) or is not (15) an independent predictor of their long-term mortality. In our MINOCA and MI-CAD groups, a diagnosis of active cancer made before index MI was more common. This overrepresentation of neoplastic status was independently associated with unfavorable long-term survival. When compared with the available literature, such a high proportion of cancer patients is primarily a result of the structure of our center, as well as that of direct admissions from oncology and thoracic surgery departments to the cardiology ward. Interestingly, there is a visible trend toward more frequent admissions of cardio-oncology patients due to their prolonged survival time.

The etiology of MI in the oncological population is multifactorial. In previous studies, the role of cancer-induced immunological disorders, oxidative stress, prothrombotic state, and oncological treatment was underlined in MI development among cancer patients (32). Moreover, oncological patients are generally high-risk due to the significant prevalence of traditional cardiovascular risk factors, such as older age, hypertension, dyslipidemia, diabetes, obesity, or tobacco addiction (28). This was also corroborated in this current study. Most of the above-indicated factors contribute to the shifted oxidase-reductase balance and endothelial injury. This exacerbates coronary artery disease progression and promotes the rupture of atherosclerotic plaque associated with type I of MI, identified as MI-CAD (28, 32, 33) in our study. On the contrary, the influence of cancer and antitumor treatment is undeniable among MINOCA survivors. The rupture of non-obstructive plaque, distal embolization, hypercoagulable state with thrombus formation, transient artery spasm, microvascular dysfunction often caused by endothelial impairment, and supply-demand mismatch, among others, are all mechanisms responsible for MINOCA (5). It is worth noting that, each of these sequences of events might be triggered by both tumor and antineoplastic treatment (13). The classic chemotherapy drugs have been proven to damage the coronary arteries, mainly in their endothelium. Therefore, they can lead to acute thrombosis and coronary spasms (33). Drugs that particularly increase the risk of MI include fluoropyrimidines (5-fluorouracil, capecitabine, gemcitabine) and platinum compounds (33), which were also often used among the analyzed patients. Moreover, combining chemotherapeutics from different groups, especially those mentioned above, significantly increases the risk of MI (33). However, there is a lack of original reports demonstrating the relationship between chemotherapy and MINOCA. Our study provides detailed angiographic and echocardiographic characteristics of cancer patients with MINOCA, shedding light on their potential relationships. These findings might be helpful in further research dedicated for personalized treatment in this demanding group of patients.

A long-term prognosis is associated with myocardial infarct size. As we have shown, both cancer and non-cancer patients with MINOCA were characterized by a better preserved global LV function and lower peak high-sensitive troponin levels compared with the corresponding MI-CAD groups. This indirectly indicates a lower myocardial injury rate and most likely a smaller infarct size in patients with MINOCA. These findings are in line with previous data showing that, among the MINOCA population, patients with heart failure with preserved LV ejection fraction (34, 35) predominated. Post-infarction myocardial remodeling is also less frequently observed in this group. There are at least a few potential explanations for this relationship. First, the smaller myocardial infarct size is a consequence of a higher prevalence of NSTEMI in MINOCA (8). Second, cardiac magnetic resonance imaging provides evidence that, in patients with MINOCA, only small foci of necrosis are often observed, while myocardial edema is the dominant abnormality (36).

In this study, hemoglobin levels were lower in both cancer groups, compared with respective non-cancer MINOCA and MI-CAD groups. Moreover, as has been shown in our multivariable models, lower hemoglobin levels worsen long-term prognosis in the whole group, but not in the population limited to patients with MINOCA. According to criteria similar to ours, anemia at baseline was found in approximately 40% of patients in the European Cancer Anemia Survey (37). This proportion increased up to 60–70% during either anticancer treatment or cancer progression, affecting the higher overall mortality risk (38). In our cancer patients with MINOCA, lower hemoglobin levels were associated with higher baseline troponin concentrations, suggesting the possibility of anemia-induced myocardial injury (16). As has been shown previously, active cancer should be considered as a secondary cause of troponinosis that is not associated with acute coronary syndrome (39, 40). Moreover, troponin elevation was linked with a higher mortality rate, especially in patients with lung cancer (41). We have also found that the ratio of troponin T to hemoglobin was significantly higher in both cancer populations when compared to the respective non-cancer groups. Our findings are one more argument for the adoption of a higher troponin cut-off value for MI in patients with cancer (39, 40). The relatively high proportion of patients with cancer-induced anemia, also visible in our cohort, may require blood transfusion or other available methods of treatment (erythropoietin or iron supplementation). In a propensity-matched analysis, Salisbury et al. have demonstrated that blood transfusion was associated with a lower risk of in-hospital mortality (42). In turn, a meta analysis done by Chatterjee et al. indicates that a liberal blood transfusion strategy is associated with higher all-cause mortality when compared to a more restricted strategy, which might be associated with volume overload, increased thrombogenicity, impaired oxygen delivery, and a risk of infection (43).

### Limitations

Our study has several limitations. First, the analyzed cancer MINOCA group is relatively small. However, it represents a unique and comprehensively characterized cohort. Second, despite their obvious heterogeneity and applied various methods of anticancer treatment, due to the small sample size of patients with different types of cancer, a multivariable analysis had to be performed for all patients with cancer. Third, cardiac magnetic resonance and intracoronary imaging were not performed to confirm an alternative diagnosis including myocarditis (44, 45). Fourth, apart from death, we did not analyze other clinical outcomes, such as recurrent MI, ischemic stroke, or heart failure decompensation. Moreover, the fact of quitting smoking after the cancer diagnosis undoubtedly contributed to its underestimated self-reporting. Finally, we also did not perform specific coagulation tests that would determine the role of prothrombotic states involved in the etiology of MINOCA (8, 46, 47).

## Conclusions

Our findings provide evidence that active cancer in the whole cohort of patients with MI, overrepresented among the MINOCA population, is associated with extremely high long-term mortality. A multivariable approach indicates that an active malignancy was independently associated with unfavorable long-term survival in the whole MI population as well as in patients with MINOCA.

## Data Availability Statement

The raw data supporting the conclusions of this article will be made available by the authors, without undue reservation.

## Ethics Statement

The studies involving human participants were reviewed and approved by Jagiellonian University Medical College Ethics Committee (Consent No. 1072.6120.59.2018). The patients/participants provided their written informed consent to participate in this study.

## Author Contributions

KS conceived the concept of the study. KS, KN, and JZ contributed to the design of the research. KS, KN, and BS reviewed the literature and were involved in data acquisition. All authors analyzed and interpreted the data. JN and JZ supervised data processing. All authors edited and approved the final version of the manuscript.

## Conflict of Interest

The authors declare that the research was conducted in the absence of any commercial or financial relationships that could be construed as a potential conflict of interest.

## Publisher's Note

All claims expressed in this article are solely those of the authors and do not necessarily represent those of their affiliated organizations, or those of the publisher, the editors and the reviewers. Any product that may be evaluated in this article, or claim that may be made by its manufacturer, is not guaranteed or endorsed by the publisher.
