# Supplementary material for: Clinical Characteristics and Long-Term Outcomes of MINOCA Accompanied by Active Cancer: A Retrospective Insight Into a Cardio-Oncology Center Registry
Source: Front Cardiovasc Med. 2022 May 20;9:785246. doi: 10.3389/fcvm.2022.785246 (PMC9163819; doi:10.3389/fcvm.2022.785246)
Supplement: Supplementary file 1 [file Data_Sheet_1.docx]

Supplementary Table 1. Baseline characteristics of cancer and non-cancer patients matched for gender, age, body mass index, diabetes, hypertension and dyslipidemia.

|  | Cancer  N=134 | Non-cancer N=524 | P-value |
| --- | --- | --- | --- |
| Male gender | 96(71.6) | 363(69.2) | 0.28 |
| Age, years | 73(65-79) | 74(66-79) | 0.33 |
| Body mass index, kg/m^2^ | 25.8(23.0-28.7) | 26.4(24.2-29.4) | 0.10 |
| Diabetes mellitus | 47(35.1) | 193(35.6) | 0.71 |
| Hypertension | 112(83.6) | 468(89.3) | 0.07 |
| Dyslipidemia | 85(63.4) | 371(72.7) | 0.10 |

Abbreviations: data are shown as number (percentage) or median (interquartile range)

Supplementary Table 2. The Cox proportional hazard regression to determine independent predictors of long-term all-cause mortality in patients matched for gender, age, body mass index, diabetes, hypertension and dyslipidemia.

|  | Univariable model | | | Multivariable model | | |
| --- | --- | --- | --- | --- | --- | --- |
|  | P-value | HR | 95% CI for HR | P-value | HR | 95% CI for HR |
| Active cancer, yes/no | <0.001 | 2.65 | 2.08-3.38 | <0.001 | 1.78 | 1.34-2.37 |
| Male gender, yes/no | 0.60 | 1.06 | 0.84-1.35 | - |  |  |
| Age, per year | <0.001 | 1.04 | 1.03-1.05 | <0.001 | 1.04 | 1.03-1.06 |
| Body mass index, per 1 kg/m^2^ | 0.18 | 0.98 | 0.95-1.01 | 0.91 | 1.00 | 0.97-1.03 |
| Diabetes mellitus, yes/no | 0.04 | 1.27 | 1.01-1.60 | 0.17 | 1.18 | 0.93-1.51 |
| Hypertension, yes/no | <0.001 | 0.49 | 0.36-0.67 | <0.001 | 0.49 | 0.35-0.69 |
| Dyslipidemia, yes/no | <0.001 | 0.53 | 0.42-0.67 | <0.001 | 0.67 | 0.52-0.86 |
| Hemoglobin, per 1 g/dl | <0.001 | 0.79 | 0.75-0.84 | <0.001 | 0.86 | 0.81-0.91 |
| LVEF, per 5% | <0.001 | 0.97 | 0.96-0.98 | <0.001 | 0.98 | 0.97-0.99 |

Abbreviations: CI: confidence interval, HR: hazard ratio, LVEF: left ventricular ejection fraction.
